# Noncommunicable Diseases and Global Health Security: Scaling up Action in Humanitarian Crises for Sustainable Recovery

**DOI:** 10.5334/aogh.4788

**Published:** 2025-06-06

**Authors:** Téa E. Collins, Amanda Karapici, Daria Berlina

**Affiliations:** 1 World Health Organization, Avenue Appia 20, 1211 Geneva, Switzerland

**Keywords:** noncommunicable diseases, humanitarian settings, health, emergencies, humanitarian crises, NCDs

## Abstract

Emergencies significantly disrupt health systems and hinder regular and timely access to service delivery. Humanitarian assistance during crises tends to prioritize populations’ immediate needs, such as injuries and infectious diseases, rather than noncommunicable diseases (NCDs), which require continuous lifelong care across different specialties and levels of healthcare systems. However, neglecting NCDs in emergency settings can have long-term negative consequences for affected populations, including increased morbidity, unnecessary preventable deaths, and straining already stressed healthcare systems even more.

The purpose of this paper is threefold. First, it argues for high-level commitments to integrate NCDs into the Global Health Security Agenda (GHSA) for more effective action toward sustainable recovery. Second, it advocates for a well-coordinated integrated health systems response that holistically tackles NCDs in humanitarian emergencies, focusing on the importance of strengthening pre-crisis infrastructure, quality NCD surveillance data, health workforce, and overall health system readiness. Finally, the paper explores the challenges for effective NCD management, emphasizing the need for multisectoral collaboration, partnerships, and resource mobilization to enhance preparedness, response, and recovery efforts.

## Introduction

The world has been facing a growing number of emergencies over the past decade, with devastating health consequences. Emergencies refer to natural disasters (e.g. earthquakes, floods, and other severe meteorological events), as well as human‑made crises, such as civil disruption, wars, chemical or radio‑nuclear spills, and infectious disease outbreaks [1]. Many emergencies are complex, involving multiple causes and impacting many people, leading to increased morbidity and mortality and damage to essential public health infrastructure and social protection systems for vulnerable populations. Most humanitarian crises occur in low‑ and middle‑income countries (LMIC). These countries often host either internally displaced people or refugees from neighboring countries, adding strain to their already overburdened healthcare systems, resulting in an increased need for funding and expanded services to meet the growing demand [2].

In 2023, the number of refugees reached 36.4 million, with more than 50% originating from Afghanistan, Syria, and Ukraine [3]. Acute food insecurity affected 258 million individuals in 58 countries, driven by armed conflicts, economic shocks, extreme weather events, poverty, and inequality. Disease outbreaks such as cholera were reported in 30 countries, due to insufficient access to clean water and sanitation, strained health systems, shortages of the oral cholera vaccine, and the presence of multiple concurrent disease outbreaks [4]. More than 1 in 73 people are forcibly displaced by conflict, a number that has almost doubled in the last 10 years [3]. In Gaza, a staggering 1.5 million individuals have experienced mass displacement, with 350,000 individuals suffering from noncommunicable diseases (NCDs) and a profound mental health burden impacting all those subjected to bombardment and siege [5].

In 2024, it is estimated that nearly 300 million people will need humanitarian assistance and protection, due to conflicts, climate emergencies, and other drivers [3]. Climate phenomena are expected to exacerbate numerous social and environmental risk factors for mental health, worsening the impacts of climate change and subsequent health challenges [6–10].

Traditionally, humanitarian responses to emergencies focused on acute conditions, such as trauma and infectious diseases, as interventions are usually prioritized according to the population’s immediate needs and resources. However, accumulating evidence indicates that in addition to the acute conditions directly related to the crisis, the risk of exacerbating pre‑existing chronic conditions, complications from multiple comorbidities, and the psychological consequences of emergencies also increase [11].

For instance, a study of young men from West Africa who fled their countries seeking asylum in Europe found that 31% of them suffered from post‑traumatic stress disorder, and 20% were diagnosed with depression [12]. Similarly, an Austrian study demonstrated that the prevalence of NCDs and mental health disorders, such as cardiovascular diseases (CVDs), digestive and urogenital problems, asthma, depression, and diabetes mellitus, was higher in migrants than in the host population [13].

Emergencies are complex events with multiple causes that impact many people, resulting in excess morbidity and mortality, damage to essential public health infrastructure and disruption of health services [14]. These events are particularly challenging for individuals living with NCDs. Damage to vital public health infrastructure and disruptions to health services can significantly intensify the risk of experiencing a sudden worsening or complication of their condition, potentially resulting in more severe long‑term prognoses or even death [14–17]. Yet, NCDs are still not part of the Global Health Security Agenda (GHSA) and were not included in the recent changes made to the International Health Regulations [18] during the 2024 World Health Assembly.

Given the persistent and increasing burden of NCDs amid various global emergencies, this paper focuses on the three major areas of action: (i) the need for high‑level commitments to include NCDs into the global health security agenda for stronger action for sustainable recovery; (ii) the importance of a well‑coordinated integrated health systems response that holistically tackles NCDs in humanitarian emergencies, focusing on strengthening pre‑crisis infrastructure, quality NCD surveillance data, health workforce, and the overall health system readiness; and (iii) the challenges for effective NCD management, and solutions to overcome these challenges through multisectoral collaboration, partnerships, and resource mobilization to enhance preparedness, response, and recovery efforts.

## NCDs in Emergencies: A Neglected Global Health Challenge

Due to their increasing prevalence, NCDs are becoming a major public health threat in emergencies, particularly in LMICs, where NCDs and humanitarian crises often intersect. However, people living with NCDs face magnified health challenges as opposed to the rest of the population during emergencies due to their chronic conditions and the need for continuous care.

The COVID‑19 pandemic is a recent example of how service disruptions disproportionately affected people living with NCDs, exacerbating their conditions and vulnerability during such crises. The 2021 World Health Organization (WHO) global survey on the national capacity for the prevention and control of NCDs [19], found that, during the pandemic, most countries experienced disruptions in various NCD services such as diabetes care (62% of 194 countries), cancer screening (59%), hypertension care (58%), and cancer therapy (53%). In the United States, 40.9% of adults avoided medical care due to COVID‑19 concerns, increasing risks for treatable conditions [20]. In Mexico, the pandemic led to a loss of millions of patient visits, impacting cancer screenings, vaccinations, and NCD consultations [21]. India also faced disruptions in providing NCD‑related medications due to the lack of service availability [22].

Due to the rapid spread of COVID‑19 worldwide, the ability of countries to address and respond to NCDs has been affected. Several examples demonstrate the significant impact of the COVID‑19 pandemic on NCD resources and services. The results of a rapid assessment conducted by WHO [23] identified the reasons for the disruption, which are summarized in Table 1.

**Table 1:** Main causes of disruption to NCD‑related services.

DISRUPTION CAUSE (BY DECREASING PREVALENCE)	% OF COUNTRIES (OUT OF 122 REPORTING DISRUPTIONS)
Decrease in inpatient volume due to cancellation of elective care	65
Closure of population‑level screening programs	46
Government or public transport lockdowns hindering access to health facilities for patients	43
NCD‑related clinical staff deployed to provide COVID‑19 relief	39
Closure of outpatient disease‑specific consultation clinics	34
Insufficient Personal Protective Equipment (PPE) for healthcare providers to provide services	33
Insufficient staff to provide services	32
Closure of outpatient NCD services as per government directive	26
Decrease in outpatient volume due to patients not presenting	25
Inpatient services/hospital beds not available	25
Unavailability/stock out of essential medicines, medical diagnostics, or other health products at health facilities	20
Others	18

*Adapted from “The impact of the COVID‑19 pandemic on noncommunicable disease resources and services: results of a rapid assessment,” World Health Organization* [23].

The data highlight critical disruptions in the delivery of NCD care, with over 75% of countries (out of 122 countries), which resulted in more advanced stages of diseases and unfavorable health outcomes [23]. The decrease in inpatient volume resulting from the cancellation of elective care underscores the challenges in resource allocation and the strain on health systems to sustain essential care, highlighting the vulnerability of the health system. Disruptions in preventive care, such as health screenings, lead to delayed diagnoses, contributing to poorer health outcomes and a backlog of undiagnosed NCDs. This emphasizes the need for resilient health systems that are capable of sustaining access to essential health services.

Addressing the burden of NCDs is particularly challenging when NCD prevalence is already high in populations before the emergency. For instance, before the start of the Syrian conflict in 2011, 77% of all deaths in Syria were attributed to NCDs, where more than 44% of this mortality was due to CVDs [24]. Accordingly, refugees from Syria living in Lebanon reported a high burden of NCDs and disabilities, with hypertension being the most common (60%), followed by diabetes mellitus (47%), and heart disease (30%) [25]. Similarly, NCDs were the leading cause of premature death (deaths between the ages of 30 and 69) in Ukraine before the crisis, accounting for 91% of the total mortality [26]. Among NCDs, CVDs were responsible for 66% of deaths, while cancers were the second leading cause at 13%. The situation is expected to worsen due to disrupted services, deteriorating living conditions, and four million people being displaced from their homes [27].

## Areas for Action

- ***High‑level commitments on NCDs and health equity***
  The current initiatives under the GHSA are predominantly focused on communicable diseases in LMICs [28], with limited integration of NCDs, thereby creating a considerable gap in comprehensive health emergency preparedness. This lack of integration is particularly concerning given the high prevalence of NCDs in LMICs, where the double burden of disease can exacerbate both health outcomes and economic instability.
  Despite considerable political and financial support, the GHSA has been criticized for emphasizing national security over individual health, with resource allocation often skewed toward high‑income countries [29], where concerns about potential disruptions to global supply chains and economic stability are more pronounced. The anticipated economic burden of NCDs, estimated to reach US$ 47 trillion by 2030 [30], emphasizes the pressing need to incorporate NCD management into global health security frameworks to ensure comprehensive emergency preparedness and response.
  The United Nations General Assembly High‑level Meeting on pandemic prevention, preparedness, and response acknowledged the growing burden of NCDs and their important role in shaping global health outcomes [31]. While this recognition is a step forward, more explicit integration of NCDs is needed. Setting clear, measurable targets and action for NCD management in health emergency plans will bridge the gap and align economic and health priorities effectively. As we approach the fourth High‑level Meeting of the UN General Assembly on the prevention and control of NCDs in 2025, stronger language is required from this meeting regarding the intersection of NCDs and emergency agendas.
  Protecting the world from infectious disease threats requires national governments’ action to ensure that those most in need, irrespective of their location or socio‑economic status, are provided with the necessary support [29]. This global responsibility should extend to individuals living with NCDs, who are disproportionately affected during health emergencies. Addressing NCDs as part of the GHSA is essential to ensure health equity.
- ***A tailored health system response***
  Strengthening health systems is essential in both emergency and non‑emergency settings to ensure continuity and quality of care for people living with NCDs. A distinguishing feature in emergencies is the urgent need to sustain life‑saving NCD services despite disrupted infrastructure, requiring adaptable delivery models and prioritized care. Emergencies strain health systems by disrupting their ability to provide services to meet the increasing demand and address risks. Consequently, health systems cannot function properly, resulting in increased morbidity and mortality. The Ebola outbreak and the COVID‑19 pandemic are just two examples that highlighted the need for resilient health systems that can adapt and maintain core functions when emergencies strike [32, 33].
  During emergencies, interruptions in healthcare, changes in healthcare priorities, reduced access to healthcare facilities, alterations in living conditions affecting diet and physical activity, higher risk for pre‑existing conditions, physiological responses to stress, medication shortages, supply chain disruptions, and other injuries or infections can lead to increased illness and death related to NCDs [34].
  NCD care in emergency settings requires a health systems response that follows a set of principles and actions tailored to the specific context and addresses both the immediate and long‑term needs of the affected population, as well as understands the baseline information about the burden of NCDs, including mortality, morbidity, and prevalence of risk factors, the level of pre‑existing NCD services, and the remaining capacity of the health system [35].
  In the emergency preparedness phase, basic information about NCDs helps prioritize which NCDs and their complications should be part of the emergency response. It also involves assessing the availability of medicines and services, estimating current and future NCD care needs, and monitoring care quality. This is vital for planning the appropriate treatment of acute complications in emergencies, including setting up a referral system [34].
  The pre‑crisis public health infrastructure and readiness of health systems to respond to emergencies and the nature of the crisis (conflict vs natural disaster or a disease outbreak) may determine the extent of disruption in health systems’ performance during emergencies, as shown by COVID‑19 [23]. Leveraging the existing resources of the health system is crucial for improving and maintaining the response to NCDs as effective management will depend on the building blocks of the health system, including health information systems, medicines, and technologies, service delivery, financial resources, governance and leadership, and especially the health workforce, which is the front line of protection against emergencies.
  Crises involving population displacement particularly strain host countries’ health systems. When Syrian refugees sought refuge in Lebanon, Turkey, and Jordan, these countries’ health systems were challenged to meet the diverse needs of the refugees, including providing continuous care for NCDs and mental health conditions, while maintaining quality services for their citizens [36]. To effectively respond to these challenges, the health system should use primary health care (PHC) as the main platform for delivering integrated and comprehensive NCD services, ranging from prevention to palliative care. It should also address the social determinants of health and the community’s mental health needs during emergencies. Additionally, adopting a people‑centered approach is essential to ensure access to quality and affordable care for NCDs, especially for the most vulnerable and marginalized groups, including women, children, the elderly, people with disabilities, and those in hard‑to‑reach areas.
- ***The need for multisectoral coordination, partnerships, and resource mobilization***
  Crises often expose populations to poor living conditions, including a lack of clean water and sanitation, limited access to employment, education, and healthcare, and food insecurity. However, few synergistic interventions are specifically aimed at mitigating these effects.
  Successful emergency response and the post‑emergency rebuilding of healthier and more resilient communities require the coordinated efforts of a wide range of stakeholders from health and non‑health sectors (the whole‑of‑government and whole‑of‑society approach) during all phases of emergency management [37].
  However, during and post‑crises, there is often a fragmented approach to coordination, especially for the NCD response, highlighting the need for greater advocacy and awareness regarding NCD care in emergencies. To tackle NCDs and mental health during humanitarian crises, well‑coordinated approaches are needed for building local capacities and leveraging international and local resources for sustainable recovery. NCDs and emergencies should not be regarded as separate agendas, but as converging challenges that require integrated and coordinated actions across sectors and stakeholders. However, additional resources will be needed to effectively integrate these agendas.
  The COVID‑19 pandemic has demonstrated the need for a renewed focus on stronger institutional arrangements and resources at global, regional, and country levels to ensure an effective response and recovery from the crises. As countries explore improved strategies for funding their healthcare systems and “building back better” initiatives for enhancing populations’ resilience, external donor support will be important to complement domestic budgets, particularly in LMICs [38].
  One of the common mechanisms for resource mobilization is multi‑partner trust funds, which pool funding from various donors and improve coordination and policy dialogue, as well as transparency of contributions. These trust funds serve as both a resource mechanism tool and a platform for collaboration and alignment among stakeholders, such as governments, donors, civil society, and the private sector. By leveraging diverse resources and expertise, multi‑partner trust funds ensure a comprehensive and sustainable approach to global health challenges. However, it is essential to implement integrated and coordinated approaches that align with the existing health systems and priorities, avoiding fragmentation and the creation of new vertical programs in global health.

Engaging with the private sector can bring benefits by leveraging their expertise, technology, and financial resources to support health initiatives. For instance, the collaboration between Gavi, the Vaccine Alliance, and various pharmaceutical companies has significantly improved access to vaccines in low‑income countries [41]. A similar model could be used for NCD care by forming partnerships with pharmaceutical and medical technology companies to ensure the availability of essential medicines and health technologies in emergencies.

Box 1. Multi‑Partner Trust Fund Examples^*^

The Health4Life Fund (H4LF) was established in 2021 by the WHO, United Nations Development Programme (UNDP), and United Nations Children’s Fund (UNICEF) following the third high‑level meeting on NCDs. The H4LF is overseen by a Steering Committee composed of UNDP, UNICEF, WHO, the UN Multi‑Partner Trust Fund Office (acting as an ex‑officio member to facilitate investor relations), civil society representatives from the NCD Alliance and United for Global Mental Health, and the governments of Kenya, Thailand, and Uruguay. The H4LF accelerates national investment in NCD prevention and mental health, promoting collaboration among governments, the UN system, and development partners while upholding evidence‑based policies and sustainable financing principles. Similarly, the United Nations Multi‑Partner Trust Fund Office administers a broad portfolio of inter‑agency pooled funds, representing a multi‑stakeholder partnership with a specific thematic and geographical focus [39].

The World Bank also administers several multi‑partner trust funds, such as the Health Emergency Preparedness and Response Multi‑Donor Fund and the Pandemic Emergency Financing Facility, playing a critical role in providing emergency financial assistance and support for health system strengthening [40].

*Health4Life Fund. 2024 Annual Report: The United Nations Multi‑Partner Trust Fund to Catalyze Country Action for Non‑Communicable Diseases and Mental Health. Published May 22, 2025. Available at: https://uniatf.who.int/health4life-fund.

Additionally, innovative financing mechanisms such as health impact bonds (HIBs) are emerging as viable options to mobilize resources for health. These instruments can improve healthcare outcomes and support underfunded initiatives such as preventive care. They provide upfront capital for health interventions with returns linked to the achievement of specific health outcomes, attracting private investments into public health [42].

In addition to financial resources, mobilizing human resources is crucial, especially considering that most actions to manage the risks to health in emergencies, including immediate emergency response, are provided by the local and national health workforce. Therefore, training and deploying health workers, including community health workers, can ensure the continuity of care during emergencies. International agencies and NGOs can help to strengthen and support local health systems by offering skills, training, and operational support.

## Conclusion

Managing NCDs in a humanitarian setting requires a high‑level political commitment and a comprehensive health systems response that adapts to the specific context and addresses both the immediate and long‑term needs of affected populations as part of emergency preparedness and response. Effective NCD management during emergencies centers around baseline information about NCD burden, pre‑crisis service levels, and the remaining capacity of health systems. This response should focus on the most urgent NCDs, make sure that essential medicines and services are available, and monitor the quality of care. Since NCD services are often disrupted during crises, as seen during the COVID‑19 pandemic, it is necessary to use existing health system resources and adopt a people‑centered approach. The response should use PHC as the platform for comprehensive NCD services and address social determinants of health. Coordinated efforts across multiple sectors and partnerships are essential for an effective and sustainable response and recovery, ensuring fair access to care for the most vulnerable populations, together with financial and human resources.
